# Antioxidant, Cytotoxic, Genotoxic, and DNA-Protective Potential of 2,3-Substituted Quinazolinones: Structure—Activity Relationship Study

**DOI:** 10.3390/ijms22020610

**Published:** 2021-01-09

**Authors:** Jana Hricovíniová, Zuzana Hricovíniová, Katarína Kozics

**Affiliations:** 1 Cancer Research Institute BMC, Slovak Academy of Sciences, Dúbravská cesta 9, 845 05 Bratislava, Slovakia; jana.hricoviniova@savba.sk; 2 Institute of Chemistry, Slovak Academy of Sciences, Dúbravská cesta 9, 845 38 Bratislava, Slovakia; chemhric@savba.sk

**Keywords:** quinazolinones, antioxidant potential, genotoxicity, cytotoxicity, structure—activity relationships

## Abstract

The evaluation of antioxidant compounds that counteract the mutagenic effects caused by the direct action of reactive oxygen species on DNA molecule is of considerable interest. Therefore, a series of 2,3-substituted quinazolinone derivatives (Q1–Q8) were investigated by different assays, and the relationship between their biological properties and chemical structure was examined. Genotoxicity and the potential DNA-protective effects of Q1–Q8 were evaluated by comet assay and DNA topology assay. Antioxidant activity was examined by DPPH-radical-scavenging, reducing-power, and total antioxidant status (TAS) assays. The cytotoxic effect of compounds was assessed in human renal epithelial cells (TH-1) and renal carcinoma cells (Caki-1) by MTT assay. Analysis of the structure–activity relationship disclosed significant differences in the activity depending on the substitution pattern. Derivatives Q5–Q8, bearing electron-donating moieties, were the most potent members of this series. Compounds were not genotoxic and considerably decreased the levels of DNA lesions induced by oxidants (H_2_O_2_, Fe^2+^ ions). Furthermore, compounds exhibited higher cytotoxicity in Caki-1 compared to that in TH-1 cells. Substantial antioxidant effect and DNA-protectivity along with the absence of genotoxicity suggested that the studied quinazolinones might represent potential model structures for the development of pharmacologically active agents.

## 1. Introduction

Oxidative DNA damage and related mutagenic processes are associated with cellular toxicity and the initiation and progression of many diseases. Compounds that are able to reduce mutagenic or genotoxic effects represent important agents with pharmaceutical utility. Among them, quinazolines and quinazolinones have been the subject of considerable interest due to their biological activities and important therapeutic effects [1,2]. These nitrogen-containing heterocycles are often used as antimicrobial [3,4], antioxidant, anti-inflammatory [5,6,7], antimalarial [8,9], antidepressant [10], anticonvulsant, or antihypertensive agents [11,12], and many of them have manifested anticancer activity [2,13,14]. The quinazolinone pharmacophore acts as a precursor to assembly a variety of new compounds for diverse applications [15,16]. The rise of bacterial resistance to traditional antibiotics has evoked a search for new antimicrobial agents [4]. A number of quinazolinone derivatives revealed selective cytotoxicity towards specific types of cancer cells [17,18,19,20] and significant antioxidant properties [6,21,22].

Under sustained oxidative stress conditions, significant damage may occur to the cell or its organelles. Reactive oxygen species (ROS) are required for normal cell function at physiological concentrations. The overproduction of ROS results in a cellular redox imbalance, which has been associated with the progression of several pathological conditions [23,24,25]. The highly reactive hydroxyl radicals generated from hydroperoxides via transition-metal-catalysed Fenton reaction [26] and iron-dependent Haber–Weiss reaction [27] can interact with nucleic acids, lipids, and proteins. Indeed, cancer initiation and progression have been associated with oxidative stress by inactivation of DNA repair enzymes, increasing DNA mutations, strand breaks, and replication errors, thus inducing DNA damage and neoplastic cell proliferation [28,29]. The occurrence of such oxidative cellular injuries indicates a deficiency of the cellular defence antioxidants such as superoxide dismutase (SOD), glutathione peroxidase (GPx), catalase (CAT), ascorbic acid, α-tocopherol, and glutathione in these particular conditions. Antioxidants may prevent potential attacks on cellular structures and protect them against oxidative damage by inhibiting reactions with ROS [30,31].

The correlation of certain diseases with the oxidative processes in vivo, induced by ROS formation, has led to an increased interest in antioxidant compounds. Biologically active compounds of natural and synthetic origin have attracted growing attention due to their radical-scavenging ability and induction of cellular antioxidant enzymes. The antioxidant activity of a particular compound depends primarily on the chemical structure, steric arrangement, the number of hydroxyl groups, and their position in the molecule. These structural features, as well as presence of aromatic rings, keto group etc., are important for the binding of hydroxyl, peroxyl, and peroxonitrite radicals. Taking into consideration the expanded applications of quinazolinone-based compounds in pharmaceutical chemistry, a series of C-2, N-3-disubstituted quinazolinone derivatives (QDs) were screened as novel safer antioxidants and potential DNA-protective agents. Introducing the phenyl rings bearing various functional groups to a quinazolinone moiety was expected to affect the antioxidant activity and to alter the biological activity.

**Scheme 1:**
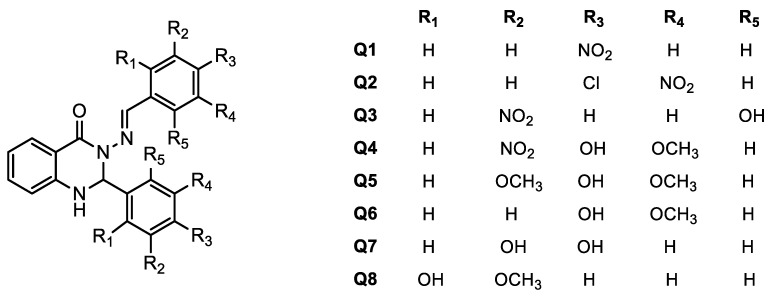
The chemical structure of C-2, N-3-disubstituted quinazolinone derivatives (Q1–Q8).

In this study, we have evaluated the potential protective effect of eight structurally different 2,3-disubstituted-2,3-dihydro-quinazolin-4-one-derived Schiff bases (Scheme 1) against hydrogen peroxide (H_2_O_2_)-induced DNA damage in human renal TH-1 cells. Furthermore, we have investigated the effect of QD treatment on the activity of enzymatic antioxidants (SOD, GPx, CAT) and the total antioxidant status (TAS) in TH-1 cells.

The radical-scavenging and antioxidant activity of the studied compounds was determined by DPPH assay, iron (III)-reducing power (FRAP), and DNA topology assay. In addition, the cytotoxic activity of quinazolinone derivatives was studied against the human renal carcinoma, Caki-1, cell line and noncancerous renal epithelial TH-1 cells. The structure–activity relationship (SAR) between the biological properties and molecular structure of the studied compounds has also been examined.

## 2. Results

### 2.1. Antioxidant Activity Determination (DPPH and FRAP Assays)

Differently substituted quinazolinone derivatives, Q1–Q8, were screened for their radical-scavenging and reducing ability using DPPH and reducing-power assays. The experimental results revealed that the studied QDs exhibited notably different and concentration-dependent DPPH-radical-scavenging effects (Table 1). Compounds Q5, Q6, and Q7 exhibited the highest radical-scavenging activity in this series (66–70%), whereas Q3, Q4, and Q8 manifested lower effect (30–35%). Very small DPPH-scavenging ability was observed in the case of Q1 and Q2 (6–8%). The ability of QDs (antioxidants) to quench DPPH free radicals by hydrogen donation increased in the following order: Q2 < Q1 < Q3 < Q4 < Q8 < Q6 ≈ Q5 ≈ Q7.

Very similar antioxidant properties of tested derivatives were identified in the FRAP assay, which measures the reducing capacity by increased sample absorbance based on the Fe^3+^/Fe^2+^ transformation by antioxidants. The QDs’ reducing power ranged between weak to high values according to their molecular structure, as seen in Table 1. Compound Q7 was the most potent reducing agent, following by Q5, Q6, and Q8. Once again, Q1 and Q2 displayed very low reducing activity. The relative reducing power of the tested QDs was found to increase in order: Q2 < Q1 < Q3 < Q4 < Q8 < Q6 < Q5 < Q7.

### 2.2. DNA Damage Protective Effect

**Figure 1:**
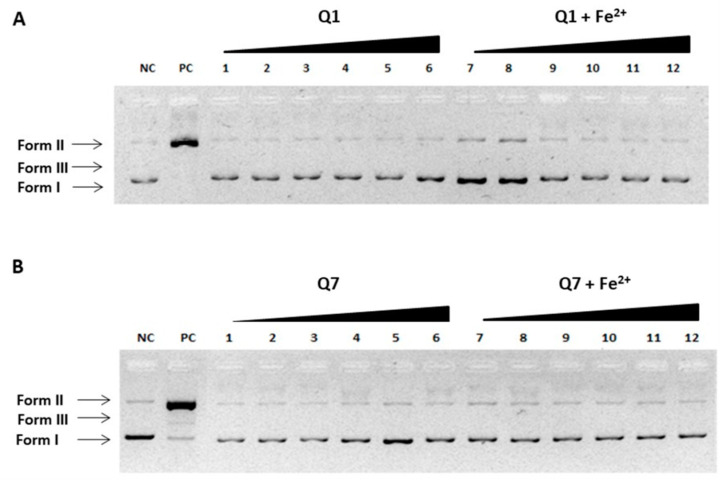
Electrophoretic monitoring of topological changes induced in the structure of plasmid DNA (pBR322) with compounds Q1 (**A**) and Q7 (**B**). Plasmid DNA treated only with Q1 and Q7 in selected concentrations (5, 10, 20, 50, 100, 500 µM) (lanes 1–6); treatment with increasing concentrations (5–500 µM) of Q1 and Q7 in the presence of Fe^2+^ ions (lanes 7–12); negative control (NC): intact plasmid pBR322; positive control (PC): pBR322 treated with Fe^2+^ ions. Treatment of pBR322 with Fe^2+^ ion-induced DNA breaks resulting in conversion of the plasmid topology from the supercoiled (form I) to single-strand breaks (form II) or double-strand breaks (form III).

The protective and/or damaging effects of the tested derivatives on DNA oxidative damage induced by Q1–Q8 and Fe^2+^ ions were evaluated by DNA topology assay. The treatment of pBR322 plasmid DNA with Q1–Q8 did not change the mobility of the supercoiled DNA indicating the non-genotoxic effect of tested compounds. Furthermore, all studied QDs were able to protect plasmid DNA against Fe^2+^-induced oxidative damage. The representative examples of two structurally very different derivatives Q1 and Q7 are shown in Figure 1A,B. Treatment with compounds Q1 and Q7 did not induce any strand breaks of the supercoiled DNA (Figure 1A,B, lanes 1–6). Moreover, only weak bands on agarose gel indicating DNA breaks are visible in two lowest concentrations (5 and 10 µM) of Q1 (Figure 1A, lanes 7–8) in the presence of a damaging agent (Fe^2+^). No visible changes in DNA topology were detected in all tested concentrations (Figure 1B, lanes 7–12) for Q7, indicating significant bio-protective ability. Similarly, treatment with Q2–Q6 and Q8 did not have any genotoxic effect on plasmid DNA, as well as on DNA-protective activity, in the presence of damaging agent (data not shown). Experimental results showed that derivatives Q1–Q8 exhibited strong DNA-protective effect against oxidative damage.

### 2.3. Cytotoxicity of Quinazolinone Derivatives (MTT Assay)

**Figure 2:**
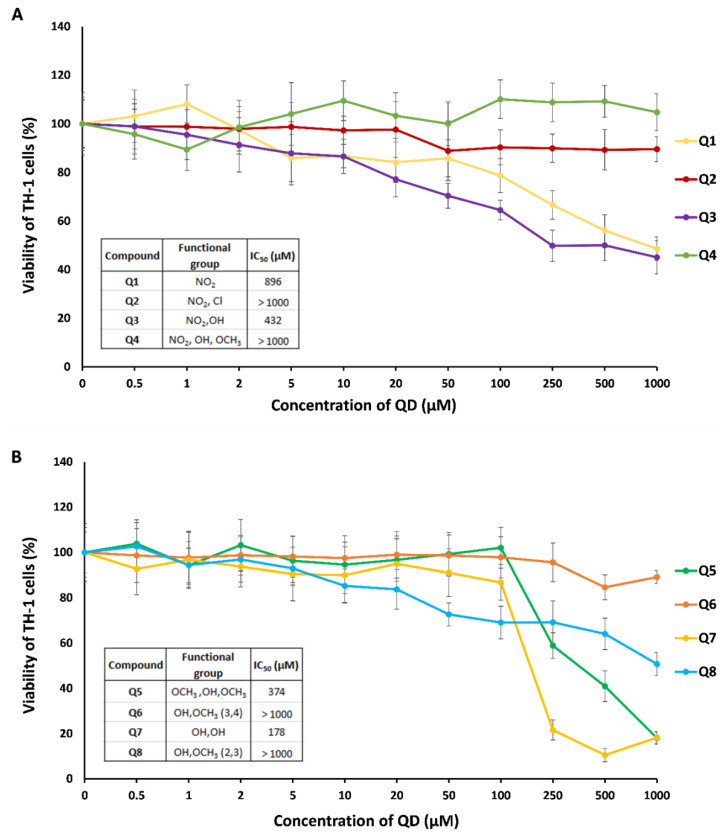
The cytotoxic effects of the studied quinazolinones on TH-1 cell viability after 24 h incubation; compounds Q1–Q4 (**A**); compounds Q5–Q8 (**B**).

**Figure 3:**
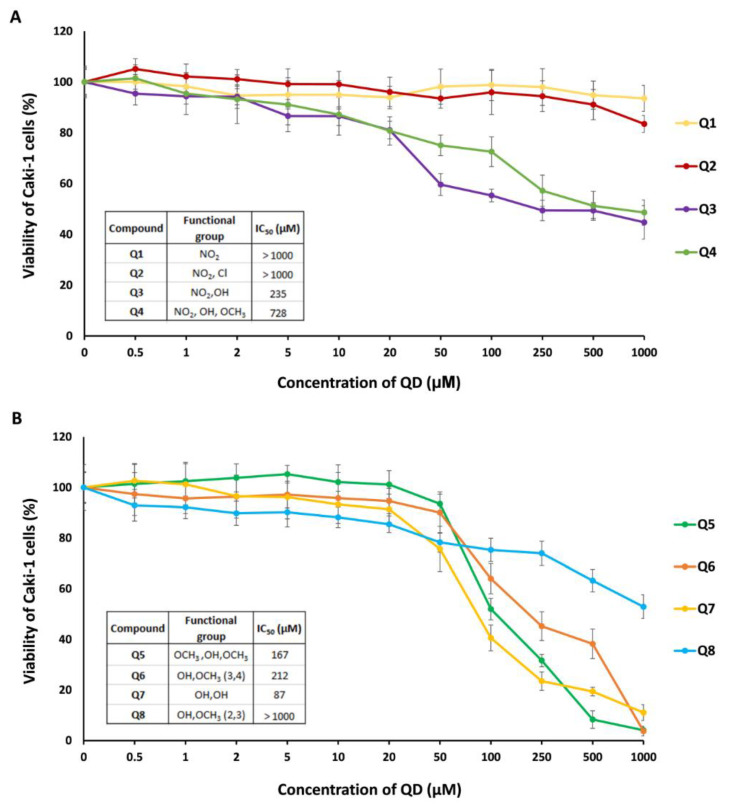
The cytotoxic effects of studied quinazolinones on Caki-1 cell viability after 24 h incubation; compounds Q1–Q4 (**A**); compounds Q5–Q8 (**B**).

The cytotoxicity of Q1–Q8 on human renal TH-1 and Caki-1 cell lines was evaluated by MTT assay in the concentration range 0–1000 µM. The results are shown in Figure 2 and Figure 3. The curves represent the viability of cells after 24 h treatment with individual QDs. Derivatives Q2, Q4, and Q6 did not have a cytotoxic effect on TH-1 cells in the tested concentration range. However, a small decrease in the cell viability was observed for Q1, Q3, and Q8 at higher tested concentrations. Derivatives Q5 and Q7 exhibited the highest cytotoxic activity (Figure 2A,B). The following order of cytotoxic activity was determined according to IC_50_ values: Q8 < Q1 < Q3 < Q5 < Q7. Further studies aimed at the genotoxic effects of QDs were assessed at IC~_10–40._

The more pronounced cytotoxic activity of some QDs was observed for Caki-1cell line. Compounds Q1, Q2, and Q8 did not inhibit the growth of cancerous Caki-1 cells. In contrast, treatment with Q3–Q7 significantly decreased the viability of the Caki-1 cell line, with an IC_50_ of 728–87 µM, respectively. The cytotoxic activity of tested derivatives was found to increase in order: Q4 < Q3 < Q6 < Q5 < Q7, and Q7 (IC_50_ 87 µM) appeared to be the most active in this group (Figure 3A,B).

### 2.4. Comet Assay

**Figure 4:**
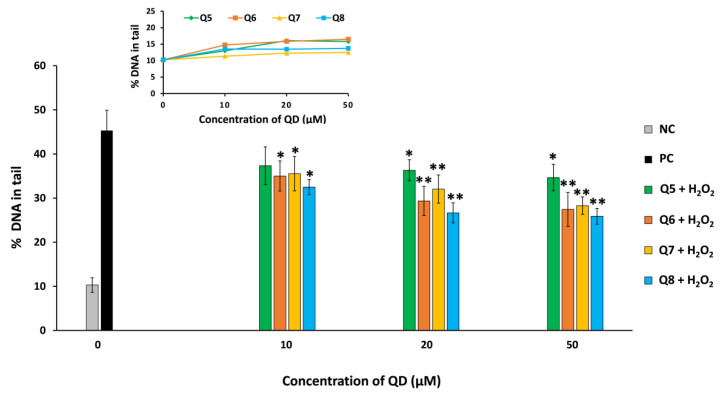
DNA-protective effect of derivatives Q5–Q8 against H_2_O_2_ measured by comet assay. TH-1 cells were pre-treated with quinazolinone derivatives (QDs) for 24 h and then treated with 500 µM H_2_O_2_ (coloured columns). TH-1 cells only with phosphate-buffered saline (PBS) were used as negative control (NC) (grey column); cells incubated only with 500 µM H_2_O_2_ correspond to a positive control (PC) (black column). The inserted panel represents the incidence of DNA damage in TH-1 cells treated with derivatives Q5–Q8 tested individually. Data represent the mean ± SD of three independent experiments. * *p* < 0.05; ** *p* < 0.01refers to significant difference to positive control.

Experimental results from the MTT assay were used for the selection of appropriate concentrations for genotoxicity assessment. The non-cytotoxic concentrations, with cell viability around 80%, were chosen for a standard comet assay. Consequently, based on the standard comet assay results, three non-genotoxic concentrations (10, 20, and 50 µM; Figure 4, inserted panel) were selected and used in experiments. For the induction of DNA single-strand breaks in TH-1 cells, H_2_O_2_ at a concentration of 500 µM was selected and used as a positive control. The higher concentration of H_2_O_2_ was used as the renal cells are more resistant to oxidative agents; 500 µM of H_2_O_2_ induced strong DNA damage corresponding to 45% of DNA in the tail.

The quinazolinone derivatives Q5–Q8 were chosen for more detailed studies, due to their strong antioxidant activity (based on DPPH and FRAP results). The assessment of the DNA-protective effect of derivatives Q5–Q8 against H_2_O_2_ on TH-1 cells was evaluated by using the comet assay (Figure 4). Experimental results showed promising DNA-protective activity of the QDs in the presence of H_2_O_2_. The tested compounds were able to reduce the level of DNA lesions in a dose-dependent manner. The quinazolinone derivatives (at 50 µM concentration) significantly decreased the levels of DNA lesions in comparison with the positive control: Q8 (43%), Q6 (40%), Q7 (38%), and Q5 (24%).

### 2.5. Effect of QDs on the Antioxidant Status and Activity of SOD, GPx, and CAT

In order to investigate the effect of pre-treatment of TH-1 cells with QDs, three non-genotoxic concentrations (10, 20, and 50 µM) of Q5–Q8 were selected for the determination of total antioxidant status and screening of antioxidant activities of individual enzymes (SOD, GPx, and CAT). The antioxidant status of Q5, Q6, Q7, and Q8 with controls is presented in Table 2. The levels of CAT and GPx in TH-1 cells pre-treated with Q5–Q8 were significantly higher in comparison with those in the negative control. The comparison of GPx and CAT levels of QDs showed that compound Q6 induced a significant increase in levels of GPx (0.031–0.035, i.e., 200–235%) at the concentration 20 and 50 µM and also exhibited the highest values of CAT activity (232.9–544.9, i.e., 63.9–283%). The SOD activity significantly increased (1.415–2.084, i.e., 47.9–117.6%) for Q6 at the concentration of 20 and 50 µM in comparison with that in the negative control. The TAS level was notably increased by Q6 (237%), Q7 (103%), and Q8 (106%) treatment, at the highest concentration (50 µM) compared to that in the negative control. Results showed that 24 h treatment of TH-1 cells with selected QDs affected the TAS, GPx, SOD, and CAT levels in a dose-dependent manner.

## 3. Discussion

Free radicals, especially ROS, have often been associated with cellular toxicity and oxidative stress that is linked with a number of harmful effects on human health. For this reason, the discovery of new and safe antioxidant compounds became necessary. In our previous study, we incorporated differently substituted phenyl rings into the quinazolinone pharmacophore [32]. The aim was to develop new compounds able to reduce the destructive effects of free radicals and protect the DNA molecule against oxidative-stress-related lesions. The properties of QDs vary considerably with even minor modifications in their molecular structure. The introduction of substituents together with their specific positions in the aromatic rings determines whether the compound behaves as an antioxidant, cytotoxic, or mutagenic agent [7,13,33]. It has been reported that substitution at positions 2 and 3 of the quinazolinone nucleus, significantly modified its biological activity and pharmacological properties. While 2-substituted quinazolinones with halogen at position 6 displayed anti-hyperlipidaemic activities, quinazolinones with 3-substitution possessed antimicrobial and antimalarial properties. However, the biological activity of 2,3-disubstituted quinazolinones has been accompanied by antioxidant, anti-inflammatory, anticonvulsant, anticancer, or antimicrobial activities [21,34,35,36].

The antioxidant activity of compounds could be related to their redox properties, which allow them to scavenge free radicals by acting as reducing agents or hydrogen donors. Their antioxidant effectiveness is related to the presence of electron-donating and/or electron-withdrawing groups in the phenyl rings attached to the quinazolinone nucleus. It is known that compounds bearing multiple hydroxyl groups in their structure are generally very good radical scavengers and reducing agents [6,37,38,39].

The radical-scavenging activities of QDs were determined by the DPPH assay. Most of the studied 2,3-disubstituted QDs exhibited a very good DPPH-radical-scavenging effect (Table 1). The data obtained for Q5, Q6, and Q7 indicate a significant antioxidant activity in comparison with ascorbic acid, which was used as a positive control. Compound Q7 exhibited the strongest radical-scavenging activity (70%) probably due to the presence of four hydroxyl groups in phenyl rings. Comparable scavenging activity (66%) was observed in the case of Q5 (4× OCH_3_, 2× OH) and Q6 (2× OCH_3_, 2× OH), especially in the higher tested concentrations. However, in the case of derivative Q8 (2× OCH_3_, 2× OH) a marked decrease (34%) of antioxidant activity was observed. Interestingly, this compound, bearing the same functional groups as Q6, displayed only moderate radical-scavenging effect, which is probably influenced by the different position of the OH/OCH_3_ group (3,4 vs. 2,3) on the phenyl rings (Scheme 1). The presence of the electron-withdrawing nitro group in Q3 and Q4 resulted in the decrease of radical-scavenging ability, and the weakest scavenging activity was observed for the compounds Q1 and Q2. The variation in the radical-scavenging effect amongst the tested Q1–Q8 derivatives was due to the different stability of the resulting oxygen-centred radical formed in these compounds. Compounds bearing electron-donating OH/OCH_3_ groups enhance the stabilization of the oxygen-centred radical, while electron-withdrawing (NO_2_ and Cl) groups destabilize the quinazoline ring. Our findings are comparable with the results of other authors published previously, studying the antioxidant and antimicrobial potency of structurally similar 2,3-disubstituted quinazolinones [6,40].

The FRAP assay may serve as a suitable method for the determination of antioxidant activity. The reduction of metal ions is used as an indicator of the electron-donating activity of compounds, thus promoting the termination of free radicals. Studied compounds Q1–Q8 exhibited different ability to reduce Fe^3+^/ferricyanide complex to Fe^2+^/ferrocyanide as seen in Table 1. Increases of reducing power were correlated with the amount of tested compound and the substitution pattern of the phenyl rings. Derivative Q7 (4× OH) showed the highest reducing effect within this series, even higher than ascorbic acid. Derivative Q5, bearing electron-donating OH and OCH_3_ groups, was the second most active. Furthermore, Q6 and Q8 having exactly the same functional groups (OH and OCH_3_), even though in different positions (3,4 vs. 2,3) on the phenyl rings, showed equally high reducing potencies. However, the presence of the electron-withdrawing substituents (Cl or NO_2_) resulted in a noticeable decrease of reducing power for derivatives Q1 and Q2. Compounds Q5–Q8 exhibited remarkable potency to donate electrons to reactive free radicals, transforming them into more stable species and terminating the free-radical chain reactions. The results shown in Table 1 demonstrated that outcomes from the FRAP assay are in good agreement with those of the DPPH assay, but the overall antioxidant activity of each tested QD can be regarded as the contribution from all the structural features in the molecule.

Plasmid DNA (pDNA) can serve as a sensitive indicator for the detection of DNA breakage. The addition of Fe^2+^ ions (oxidant) induces the formation of strand breaks in the pDNA via Fenton-like reaction, thus resulting in DNA structural changes. Incubations of pDNA with QDs did not change the mobility of the supercoiled DNA in the selected concentration range (5–500 µM), which indicates to non-genotoxic effects of all tested compounds from this series. Additionally, agarose gel electrophoresis patterns confirmed a significant degree of protective efficacy of all QDs against the Fe^2+^-induced oxidative damage. Figure 1A,B shows the electrophoretic monitoring of topological changes in DNA after the application of two structurally very different derivatives, Q1 and Q7, and their protective activity in the presence of Fe^2+^ ions. Treatment with compound Q7 (the most potent antioxidant), as well as compound Q1 (the least potent antioxidant), did not induce any strand breaks of the supercoiled DNA (Figure 1A,B, lanes 1–6). The DNA-protective effect was assessed by measuring the degree of protection against Fe^2+^-induced DNA breaks. In the presence of a damaging agent (Fe^2+^), only weak bands were visible in the two lowest concentrations (5 and 10 µM) of Q1 (Figure 1A, lanes 7–8). In the case of compound Q7, no changes in pDNA topology were detected in all tested concentrations (Figure 1B, lanes 7–12). Derivatives Q2–Q6 and Q8 showed non-genotoxic effects and displayed very similar protective activity.

The cytotoxic response of various cell lines to different compounds is generally screened by an MTT assay. This method is suitable to measure cellular metabolic activity as an indicator of cell viability and/or cytotoxicity. The cytotoxic effect of Q1–Q8 was investigated in vitro with the use of two human renal cell lines, TH-1 and Caki-1. The Caki-1 cell line was proposed as a model system of proximal tubule epithelium, as in culture, cells can form a layer with the morphological, physiological, and biochemical characteristics of functional, well-differentiated kidney tissue [41].

The results of the MTT assay showed different inhibitory activities for the studied QDs. Studied compounds affected cell viability of both TH-1 and Caki-1 cell lines cultured in vitro in a concentration-dependent manner. When evaluating the TH-1 cell viability, no cytotoxic activity was observed in the presence of Q2, Q4, and Q6. Among the tested compounds, Q7 showed the highest cytotoxic activity (IC_50_ 178 µM) while treatment with Q1, Q3, Q5 revealed only mild, and in the case of Q8, a very low cytotoxic effect. The IC_50_ values of the tested QDs revealed that the TH-1 cell line is most susceptible to the Q7 derivative (Figure 2A,B). However, MTT assays indicated that QDs displayed approximately two-fold higher inhibitory effect on the viability of renal cancer cells, Caki-1, in comparison with normal that in TH-1 cells. Compounds Q1 and Q2 with chlorine and nitro group in their structure did not affect the viability of Caki-1 cells. Interestingly, the cell viability significantly decreased in Caki-1 cells after treatment with Q5, Q6, and Q7 bearing multiple electron-donating OH and OCH_3_ groups. Additionally, in this case, Q7 (IC_50_ 87 µM) was recognized as the most efficient derivative in this series (Figure 3A,B).

Faraj and Zahedifard and their colleagues have investigated the antiproliferative activity of structurally very similar quinazolinone Schiff bases in normal MCF-10 breast cells, MCF-7 human breast cancer cells, and normal WRL-68 hepatic cells. Studied compounds significantly inhibited the viability of MCF-7 cells, but they exhibited no suppressive effect against human normal WRL-68 and MCF-10 cells [18,42]. Hassanzadeh et al. have studied the effect of quinazolinone-1,3,4-oxadiazole derivatives on the viability of MCF-7 and HeLa cervical cancer cell line and found that these compounds exhibited remarkable cytotoxicity [43]. The cytotoxic effects of structurally different QDs have been extensively studied also by other research groups using various cell lines, e.g., BV2, N2a, MCF-7, A546, HT-29, SW620. It was observed that the cytotoxicity of QDs apparently depends on the presence of a substituent located at carbon C-2 that could improve the activity of these compounds possibly due to the electronic effects. The detailed SAR analysis allows the determination of the chemical groups responsible for the desired therapeutic effects [42,44,45,46].

In the present study, the comet assay was used to demonstrate the DNA-protective effects of selected derivatives, Q5–Q8, against oxidant (H_2_O_2_) in the TH-1 cell line. Intracellular ROS generation indicated that the treatment of in TH-1 cells with H_2_O_2_ induces predominantly DNA strand breaks via the formation of hydroxyl radicals by the Haber–Weiss reaction, which is catalysed by ferric ion [27]. ROS, especially hydroxyl radicals produced in situ during the oxidative cycle, have the ability to diffuse through the cell membranes and cause undesirable cellular injury [47,48]. In the current study, the 24 h pre-treatment with QDs disclosed their protective effects against oxidant-induced DNA damage in TH-1 cells. The DNA-protective effect of Q6 and Q8 (2× OH, 2× OCH_3_) was more prominent in comparison with that of Q5 (2× OH, 4× OCH_3_) and Q7 (4× OH) as seen in Figure 4. Based on our results, it can be concluded that not only the same number of the OH and OCH_3_ groups but also their position on the quinazolinone ring is crucial for the DNA-protective effects. Our results are in very good correlation with a previous study from Zhao and Liu who reported the protective effects of hydroxyl-substituted Schiff bases against radical-induced oxidation of DNA [49].

Antioxidant compounds act through multiple mechanisms to dampen or counteract oxidative stress either by reducing the cause or the consequences of oxidative stress. Cells are protected against oxidative stress by the antioxidant network. Antioxidant enzymes SOD, CAT, and GPx serve as a primary line of defence in destroying free radicals [24,48]. Intracellular SOD and GPx present in the cytosol and mitochondria reduce the superoxide radical anion to H_2_O_2_ and water and remove the majority of H_2_O_2_ as well. In a meantime, CAT, present in peroxisomes, eliminates high levels of H_2_O_2_ as well. Therefore, antioxidant enzymes play a major role in reducing the rate of production of new ROS and are referred to as preventive antioxidant agents [30].

Experimental data showed that the treatment of TH-1 cells with compounds Q5–Q8 led to statistically significant increase of enzyme activity in comparison with that in control samples (Table 2). The level of SOD, which detoxifies superoxide radicals, was increased in the order Q8 < Q7 < Q5 < Q6, and Q6 (117%), and appeared to be the most active in this group. Furthermore, derivative Q6 also caused a significant increase in levels of CAT (64–283%) and GPx (52–235%), which disactivate H_2_O_2_ by converting it to water and oxygen. Results demonstrated that the treatment of TH-1 cells with compounds Q5–Q8 affected SOD, CAT, GPx, and TAS levels in a dose-dependent manner. Derivative Q6 (2× OCH_3_, 2× OH) considerably increased the level of all antioxidant enzymes (GPx, CAT, SOD) and TAS in comparison with Q5, Q7, and Q8.

Heterocyclic structural motifs such as quinazolinones are an integral part of many natural products with biological potential. Similarly, polyphenols present in natural plant extracts and essential oils are known for their strong antioxidant effects and may interact with intracellular antioxidant species and may enhance their antioxidant activity [39,50]. The phenolic moieties in the molecular structure of QDs probably contribute to the increased expression of intracellular antioxidant enzymes in TH-1 cells. Results obtained in this study are in very good correlation with those published by other authors [6,49] who reported that presence of hydroxyl groups on the aromatic ring makes hydroxyl-substituted Schiff bases the effective antioxidants and potential drugs to prevent diseases related to free-radical damage.

Physiological processes are very sensitive to ROS production, thus cellular redox homeostasis is crucial for the control of cell proliferation and differentiation. Interruption of the redox pathways that regulate ROS and its redox signalling can ultimately result in cancer development [28]. However, the modulation of the redox balance can be useful in cancer therapy. Compounds that elevate ROS production are able to increase the cellular ROS level additionally. This can be accomplished either by the direct ROS generation or by agents that break off the inherent antioxidant system carried by endogenous antioxidant enzymes. The overall increase in the production of endogenous ROS may induce the apoptosis of cancer cells [29].

Based on the results obtained in this study, we can conclude that the studied QDs exhibited only a modest cytotoxic effect against human renal cancer cells, but they showed strong antioxidant activity. They disrupted the electron transport chain reactions due to their remarkable radical-scavenging properties. Furthermore, they simultaneously induced an increase of antioxidant enzyme levels. In this regard, these compounds could be considered chemopreventive agents able to reduce the risk of ROS-induced oxidative damage.

## 4. Material and Methods

### 4.1. Chemicals, Materials, and Equipment

Ethidium bromide (EtBr), Triton X-100, ethylenediaminetetraacetic acid disodium salt dihydrate (Na_2_EDTA2H_2_O), trichloroacetic acid, 1,1-diphenyl-2-picrylhydrazyl (DPPH) radical, dimethyl sulphoxide (DMSO), 3-(4,5-dimethylthiazol-2-yl)-2,5-diphenyltetrazolium bromide (MTT), bovine serum albumin (BSA), glutathione reductase, glutathione, nicotinamide adenine dinucleotide reduced form (NADPH), normal-melting-point (NMP) agarose, and low-melting-point (LMP) agarose were purchased from Sigma-Aldrich Co. (Steinheim, Germany); pBR322 plasmid DNA was purchased from New England BioLabs Inc. (-Ipswitch, MA, USA); GelRed Nucleic Acid Gel Stain from Biotium (Fremont, CA, USA); phosphate-buffered saline (PBS; Mg^2+^ and Ca^2+^-free) from OXOID LIMITED (Basingstoke, UK); RANSOD kit was purchased from Randox Laboratories Ltd. (Crumlin, UK); hydrogen peroxide (Sigma-Aldrich Co., Steinheim, Germany) was kept at 4 °C and diluted in PBS to a final concentration of 500 μM before the treatment of cells. Media, antibiotics, and chemicals used for in vitro experiments were purchased from Gibco BRL (Paisley, UK).

High-resolution NMR spectra were recorded in a 5 mm cryoprobe at 25 °C on a Bruker Avance III HD spectrometer at 14 T in deuterated dimethyl sulphoxide (DMSO-d_6_). Fourier-transform infrared (FT-IR) spectra, in the range of 400–4000 cm^−1^, were measured on a Nicolet 6700 spectrometer (Thermo Fisher Scientific, Waltham, MA, USA) with a DTGS detector and OMNIC 8.0 software using 128 scans at the resolution of 4 cm^−1^ with diamond attenuated total reflectance (ATR) technique. High-resolution mass spectra (HRMS) were measured on an Orbitrap Velos PRO (Thermo Scientific, Waltham, MA, USA), with electrospray ionization method, operated in positive mode. Elemental analyses were performed on Flash 2000 CHNS/O Elemental Analyzer (Thermo Scientific, Waltham, MA, USA). Melting points were determined on a Kofler hot-stage microscope. The progress of the reactions was monitored by thin-layer chromatography (TLC) on aluminium sheets pre-coated with Silica Gel 60 F_254_ (Merck Milipore, Darmstadt, Germany). All other chemicals and solvents were of analytical grade from commercial suppliers.

### 4.2. Studied Compounds

Quinazolinone derivatives, Q1–Q8 (Scheme 1), were of high purity (99%). Compounds Q1–Q7, were synthesized by Hricovíniová et al., and all spectral data for Q1–Q7 were reported in our previous work [32]. All QDs are stable in the solid state as well as in solution. Derivative Q8 was synthesized according to Gudasi et al. [51], and its identity and purity was confirmed by spectroscopic methods and elemental analysis.

3-[(2-hydroxy-3-methoxybenzylidene)amino]-2-(2-hydroxy-3-methoxyphenyl)-2,3-dihydroquinazolin-4-one (Q8). Yellow solid, yield 87.1%; m.p. 209–210 °C; *R_f_* 0.69 (chloroform/ethyl acetate/methanol 1:1:0.5 *v/v/v*); Elemental analysis calculated for C_23_H_21_N_3_O_5_ (%): C 65.86, H 5.05, N 10.02; found: C 65.91, H 5.02, N 10.07; FT-IR (cm^−1^): 3279 (N−H), 3058 (O−H), 3042 (C−H)_Ar_, 1655 (C=O), 1606 (HC=N), 1263 (N−N), 1151 (C−N), 1036 (O−CH_3_); ^1^H NMR (DMSO-d_6_, 600 MHz) δ (ppm): 11.32 (s, 1H, O–H”), 9.41 (s, 1H, O–H’), 8.41 (s, 1H, N=C–H), 7.45 (s, 1H, N–H), 7.30−6.65 (m, 10H, H_Ar_), 3.81 (s, 3H, OCH_3_’), 3.79 (s, 3H, OCH_3_”); HRMS for C_23_H_21_N_3_O_5_Na: calc. [M + Na]^+^ 442.4197; found 442.4191; Data obtained were in good agreement with published results [51]. Compounds were kept in a dark place and dissolved in dimethyl sulphoxide (final concentration 0.5%) prior to use.

### 4.3. Methods for the Determination of Biological Activity of Quinazolinone Derivatives

#### 4.3.1. 1,1-Diphenyl-2-Picrylhydrazyl (DPPH) Assay

The radical-scavenging activity of the tested compounds was evaluated by DPPH assay according to Locatelli et al. with some modifications [52]. DPPH solution (100 μM) was prepared by dissolving DPPH radical in methanol. The synthesized compounds (Q1–Q8) at different concentrations (5, 10, 50, 100, 500 μM) were dissolved in DMSO. The reaction mixture (consisting of 190 µL of DPPH solution and 10 µL of tested derivative) was added to 96-well plate, shaken, and incubated in the dark for 30 min. After incubation, the absorbance of the resulting solution was measured at 517 nm using an xMark™ Microplate Spectrophotometer (Bio-Rad Laboratories, Inc., Hercules, CA, USA). Ascorbic acid was used as a positive control and DPPH solution was used as a negative control. The antioxidant activity percentage was evaluated using the equation:

Scavenging of DPPH radicals (%) = (A_control_ − A_sample_/A_control_) × 100

where A_control_ is the absorbance of negative control; A_sample_ is the absorbance of the tested compound.

#### 4.3.2. Iron (III)-Reducing Power (FRAP Assay)

The antioxidant and reducing capacities of the tested compounds were determined by the colorimetric reducing-power assay [53]. Different concentrations of the tested compounds, Q1–Q8 (5–500 μM), in DMSO were mixed with 0.2 M phosphate buffer (pH 6.6) and 1% potassium ferricyanide [K_3_Fe(CN)_6_]. The mixture was incubated at 50 °C for 20 min. Then, 10% trichloroacetic acid solution was added to the mixture and centrifuged at 3000 rpm for 10 min. The upper layer of the supernatant was mixed with distilled water and 0.1% FeCl_3_ solution. Afterwards, the absorbance was measured at 700 nm using an xMark™ Microplate Spectrophotometer.

#### 4.3.3. DNA Topology Assay

This method is based on the electrophoretic detection of topological changes in the plasmid DNA (pBR322) in the presence of Fe^2+^ ions [54]. Briefly, the studied quinazolinones (Q1–Q8) at different concentrations (5, 10, 20, 50, 100, and 500 μM) were dissolved in DMSO and diluted in distilled water. The reaction mixture (final volume of 10 μL) contained 1 μL of plasmid DNA (diluted in the ratio 1:30 with H_2_O), phosphate buffer (pH 7.4) (1 μL), Fe^2+^ alone (1 μL) (positive control), tested compound alone (1 μL), or combinations of the tested compound with Fe^2+^ions. The presence of Fe^2+^ ions induces the formation of free radicals resulting in topological changes of pDNA, altering its mobility in the agarose gel. DNA damage was measured as the conversion of supercoiled DNA (form I) to a relaxed (form II) (single-strand breaks). Analysis of DNA modifications was made by agarose gel electrophoresis (1% agarose, 75 min, 70 V) and visualized by staining with GelRed in the program GelCapture and UV illumination (UV Transilluminator MiniBISPro, DNR Bio Imaging Systems Ltd., Neve Yamin, Israel).

### 4.4. Cell Cultures

The human renal proximal tubule epithelial TH-1 cell line was purchased from Kerafast Inc. (Boston, MA, USA). The TH-1 cells were cultured in Dulbecco’s-modified Eagle’s medium (DMEM); human renal carcinoma cell line (Caki-1) was purchased from ATCC (Manassas, VA, USA), cultured in McCoy’s 5A Medium. Media for both cell lines were supplemented with 10% foetal bovine serum (FBS) and antibiotics (penicillin 100 U/mL, streptomycin 100 μg/mL) on Petri dishes (Ø 100 mm) at 37 °C in a humidified atmosphere of 5% CO_2_.

#### 4.4.1. Cytotoxic Activity (MTT Assay)

The cytotoxicity of compounds Q1–Q8 was monitored by the colorimetric MTT cell assay. In this assay, the yellow tetrazolium salt 3-(4,5-dimethylthiazol-2-yl)-2,5-diphenyltetrazolium bromide (MTT) is reduced by mitochondrial enzymes of metabolically active cells to purple formazan and is spectrophotometrically quantified [55]. Exponentially growing cells were pre-incubated in the presence of different concentrations (0–1000 µM) of the tested compounds for 24 h. Cells treated with the medium only served as a negative control. The properly treated TH-1 cells were incubated in 100 μL of complete medium and 50 μL of MTT solution (1 mg/mL in PBS) for 4 h. After incubation, the MTT solution was removed, the formazan crystals in each well were dissolved in DMSO (100 μL) for 30 min. The absorbance intensity was measured using an xMark™ Microplate Spectrophotometer at 540 nm with a reference wavelength of 690 nm. All experiments were performed in triplicate, and the relative cell cytotoxicity was expressed as a percentage relative to the untreated control cells. Unexposed cells were used as control and considered as having 100% of cell viability. The viability of cells was calculated by the following formula: Viable cells (%) = A_treated cells_/A_control cells_ × 100.

#### 4.4.2. Single-Cell Gel Electrophoresis (SCGE; the Comet Assay)

The assessment of DNA damage in TH-1 cells was evaluated by the alkaline comet assay [56]. Exponentially growing cells were pre-incubated in the presence of the studied QDs (non-cytotoxic concentration range for QD was chosen based on MTT assay), or without QD (control) for 24 h. In brief, an appropriate number of cells were washed, trypsinized, centrifuged (10 min, 1000 rpm), and re-suspended in 0.75% low-melting-point agarose. Approximately 2 × 10^4^ aliquots of TH-1 cells were spread on a base layer of 1% normal-melting-point agarose in PBS on microscopic slides and covered with cover slips. After solidification of the gels, the coverslips were removed, and samples were treated with 500 µM H_2_O_2_ (5 min on ice in the dark). Then samples were placed in lysis solution (2.5 M NaCl, 100 mM Na_2_ EDTA, 10 mM Tris–HCl, pH 10, and 1% Triton X-100, at 4 °C) for 1 h. Slides were transferred to an electrophoresis solution (300 mM NaOH, 1 mM Na_2_ EDTA, pH > 13) for unwinding (30 min, 4 °C) and then subjected to electrophoresis (19 V, 300 mA, 20 min, 4 °C). The slides were neutralized with Tris–HCl (0.4 M, pH 7.4) and stained with ethidium bromide (EtBr, 5 µg/mL). EtBr-stained nucleotides were examined with a Zeiss Imager, Z2 fluorescence microscope using computerized image analysis (Metafer 3.6, MetaSystems GmbH, Altlussheim, Germany). The percentage of DNA in the tail (% of tail DNA) was used as a parameter for the measurement of DNA damage (DNA strand breaks). One hundred comets were scored per sample in one electrophoresis run.

#### 4.4.3. Total Antioxidant Status (TAS) and Antioxidant Enzymes Activity Assays

The TAS was determined by the chromogenic method (Randox Laboratories, UK) with minor alterations. The method is based on the capacity of a compound to inhibit the formation of the ABTS^+^• radical cation (2,2′-Azino-bis-[3-etylbenzotiazolinesulphonate]) [57]. Absorbance at 600 nm was measured. Ascorbic acid (10 µmol/L) was used as a positive control. Results are expressed as µmol of TAS per gram of protein (µmol/g protein).

For the determination of SOD (EC 1.15.1.1) and GPx (EC 1.11.1.9) activities, treated and untreated (control) TH-1 cells were used. Cells were dissolved 1:1 in 0.1% Triton X-100. For the determination of the SOD activity of TH-1 cells (1.5 × 10^4^), the RANSOD kit was used. The method employs xanthine and xanthine oxidase to generate superoxide radicals, which react with 2-(4-iodophenyl)-3-(4-nitrophenol)-5-phenyltetrazolium chloride to form a red formazan dye. The SOD activity was determined as the degree of inhibition of this reaction measured by absorbance at 505 nm. The method of Paglia and Valentine was applied for the determination of GPx activity in the TH-1 cells (3 × 10^4^) with cumene hydroperoxide as a substrate [58]. The absorbance shift was measured at 340 nm. CAT (EC 1.11.1.6) activity was determined according to Góth [59]. Samples of TH-1 cells (5 × 10^5^) were incubated with H_2_O_2_ as a substrate at 37 °C for 1 min. The enzymatic reaction was stopped by adding ammonium molybdate to the reaction mixture. The absorbance of the yellow complex formed by molybdate and H_2_O_2_ was measured at 405 nm. The specific activity of CAT was expressed as U/mg of the protein. One unit of CAT activity was defined as the amount of enzyme that decomposes 1 μmol/L of H_2_O_2_/min. Similarly, the activities of SOD and GPx were expressed as U/mg of protein. In all experiments, an xMark^TM^ Microplate Spectrophotometer (Bio-Rad Laboratories, Inc., Hercules, CA, USA) was used. The protein concentrations were determined using the Bradford method [60].

### 4.5. Statistical Analysis

The results are presented as means from at least three sets of independent experiments ± standard deviation (±SD). The differences between defined groups were tested for statistical significance using Student’s *t*-test (* *p* < 0.05; ** *p* < 0.01; *** *p* < 0.001).

## 5. Conclusions

In summary, results from the present study indicate that the studied QDs are promising antioxidants and DNA-protective agents. The DNA-protective ability could be explained both by elevated levels of cellular enzymatic antioxidants-SOD, GPx, CAT, total antioxidant status in TH-1 cells pre-treated with QD and their self-antioxidant activity as evidenced by DPPH and FRAP assays. Compounds Q5, Q6, and Q7 exhibited higher cytotoxicity against renal cancer cells, Caki-1, compared to normal TH-1 cells. SAR analysis revealed that compounds Q5–Q8, bearing multiple electron-donating moieties, are the most efficient derivatives in this group. The comparison of biological properties supposes that the molecular structure of individual quinazolinone derivatives substantially contributes to the different efficiency of the studied compounds. However, their potential applications in pharmacology will require additional validation using further in vitro and in vivo studies.

## Figures and Tables

**Table 1:** DPPH-radical-scavenging and reducing-power assays of 2,3-disubstituted quinazolinone derivatives (Q1–Q8) in comparison with ascorbic acid (AA).

Compound		Concentration (µM)	DPPH-Radical-Scavenging Activity (%)	Fe^3+^/Fe^2+^-Reducing Power (Absorbance)
**Q1**	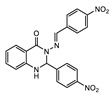	5	1.860 ± 0.954	0.022 ± 0.009
		10	2.380 ± 1.490	0.039 ± 0.033
		50	2.560 ± 1.702	0.056 ± 0.052
		100	3.680 ± 2.607	0.068 ± 0.075
		500	7.990 ± 2.115	0.075 ± 0.047
**Q2**	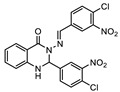	5	2.700 ± 0.799	0.013 ± 0.039
		10	3.403 ± 1.716	0.025 ± 0.014
		50	4.043 ± 0.978	0.029 ± 0.005
		100	4.941 ± 1.122	0.048 ± 0.093
		500	5.895 ± 0.981	0.057 ± 0.076
**Q3**	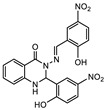	5	3.903 ± 1.721	0.058 ± 0.036
		10	5.514 ± 2.522	0.109 ± 0.083
		50	6.331 ± 2.035	0.145 ± 0.014
		100	16.300 ± 1.862	0.432 ± 0.037
		500	30.077 ± 1.991	0.817 ± 0.056
**Q4**	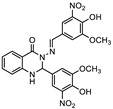	5	13.760 ± 2.175	0.055 ± 0.029
		10	23.217 ± 1.602	0.098 ± 0.071
		50	29.190 ± 2.505	0.221 ± 0.052
		100	31.980 ± 2.801	0.530 ± 0.014
		500	35.812 ± 1.734	0.899 ± 0.059
**Q5**	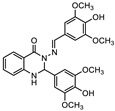	5	20.234 ± 0.094	0.071 ± 0.036
		10	25.481 ± 1.287	0.193 ± 0.051
		50	36.297 ± 0.981	0.282 ± 0.025
		100	42.642 ± 1.092	0.430 ± 0.072
		500	66.454 ± 1.185	1.174 ± 0.019
**Q6**	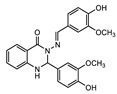	5	17.326 ± 1.667	0.061 ± 0.021
		10	20.781 ± 2.852	0.070 ± 0.006
		50	32.016 ± 0.975	0.197 ± 0.042
		100	41.546 ± 2.093	0.411 ± 0.062
		500	65.967 ± 1.881	1.017 ± 0.027
**Q7**	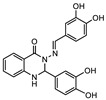	5	24.001 ± 1.813	0.087 ± 0.052
		10	29.717 ± 1.617	0.105 ± 0.048
		50	35.202 ± 1.922	0.315 ± 0.033
		100	43.342 ± 1.754	0.654 ± 0.044
		500	69.998 ± 1.079	1.386 ± 0.109
**Q8**	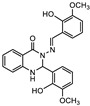	5	19.561 ± 2.002	0.078 ± 0.048
		10	21.497 ± 1.808	0.081 ± 0.072
		50	23.525 ± 1.682	0.237 ± 0.033
		100	25.726 ± 2.219	0.369 ± 0.081
		500	33.847 ± 1.762	1.027 ± 0.514
**AA**	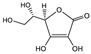	5	20.071 ± 0.993	0.125 ± 0.026
		10	25.006 ± 1.274	0.269 ± 0.007
		50	42.121 ± 2.186	0.523 ± 0.085
		100	77.820 ± 1.053	0.685 ± 0.015
		500	88.752 ± 1.743	1.259 ± 0.076

Data represent the means ± standard deviations (SD) of three independent experiments; ascorbic acid (AA) was used as a positive control.

**Table 2:** Total antioxidant status (TAS) of selected quinazolinone derivatives, Q5–Q8, and the activity of individual enzymes—superoxide dismutase (SOD), glutathione peroxidase (GPx), and catalase (CAT) in TH-1 cells after 24 h treatment.

	TAS	SOD (U/mg Prot)	GPx (U/mg Prot)	CAT (U/mg Prot)
Control (−)	0.795 ± 0.120	0.957 ± 0.070	0.0104 ± 0.0008	142.109 ± 2.290
Control (+)	3.320 ± 0.080	2.520 ± 0.520	0.039 ± 0.002	585 ± 54
**Q5**				
10 µM	0.828 ± 0.040	0.804 ± 0.210	0.0161 ± 0.0008 **	217.756 ± 16.280 *
20 µM	0.992 ± 0.025	1.471 ± 0.130	0.0187 ± 0.0028 *	237.274 ± 1.948 ***
50 µM	1.016 ± 0.060	1.537 ± 0.290	0.0229 ± 0.0023 **	285.640 ± 0.956 ***
**Q6**				
10 µM	0.755 ± 0.090	1.041 ± 0.180	0.0158 ± 0.0015 **	232.940 ± 7.437 **
20 µM	1.091 ± 0.240	1.415 ± 0.440	0.0312 ± 0.0029 **	489.357 ± 27.782 **
50 µM	2.678 ± 0.110 **	2.084 ± 0.001 *	0.0349 ± 0.0006 **	544.981 ± 29.630 **
**Q7**				
10 µM	1.033 ± 0.410	0.980 ± 0.070	0.0121 ± 0.0007	240.114 ± 3.354 ***
20 µM	1.247 ± 0.420	1.023 ± 0.180	0.0140 ± 0.0007 *	243.603 ± 16.312 **
50 µM	1.615 ± 0.110 *	1.031 ± 0.150	0.0197 ± 0.0018 **	261.402 ± 2.166 ***
**Q8**				
10 µM	0.841 ± 0.350	0.792 ± 0.140	0.0149 ± 0.0007 *	232.392 ± 6.750 ***
20 µM	1.040 ± 0.040	0.869 ± 0.010	0.0162 ± 0.0012 **	244.104 ± 1.994 ***
50 µM	1.636 ± 0.110 *	1.077 ± 0.030	0.0202 ± 0.0021 **	323.904 ± 8.911 ***

Control (−) untreated TH-1 cells; control (+) ascorbic acid. Data represent the mean ± SD of three independent experiments. * *p* < 0.05; ** *p* < 0.01; *** *p* < 0.001 indicate significant differences compared to the untreated control cells.

## Data Availability

Data is contained within the article or supplementary material.

## References

[1] Rakesh K.P., Darshini N., Shubhavathi T., Mallesha N. (2017). Biological applications of quinazolinone analogues: A Review. Org. Med. Chem. Int. J..

[2] Selvam T.P., Kumar P.V. (2011). Quinazoline Marketed drugs—A Review. Res. Pharm..

[3] Jatav V., Kashaw S., Mishra P. (2008). Synthesis, antibacterial and antifungal activity of some novel 3-[5-(4-substituted phenyl) 1,3,4-thiadiazole-2-yl]-2-styryl quinazoline-4(3H)-ones. Med. Chem. Res..

[4] Jafari E., Khajouei M.R., Hassanzadeh F., Hakimelahi G.H., Khodarahmi G. (2016). Quinazolinone and quinazoline derivatives: Recent structures with potent antimicrobial and cytotoxic activities. Res. Pharm. Sci..

[5] Hussein M.A. (2013). Synthesis, anti-inflammatory, and structure antioxidant activity relationship of novel 4-quinazoline. Med. Chem. Res..

[6] Rakesh K.P., Manukumar H.M., Gowda D.C. (2015). Schiff’s bases of quinazolinone derivatives: Synthesis and SAR studies of a novel series of potential anti-inflammatory and antioxidants. Bioorg. Med. Chem. Lett..

[7] Kumar A., Sharma S., Bajaj A.K., Sharma S., Panwar H., Singh T., Srivastava V.K. (2003). Some new 2,3,6-trisubstituted quinazolinones as potent anti-inflammatory, analgesic and COX-II inhibitors. Bioorg. Med. Chem..

[8] Zhu S., Wang J., Chandrashekar G., Smith E., Liu X., Zhang Y. (2010). Synthesis and evaluation of 4-quinazolinone compounds as potential antimalarial agents. Eur. J. Med. Chem..

[9] Birhan Y.S., Bekhit A.A., Hymete A. (2015). In Vivo antimalarial evaluation of some 2,3-disubstituted-4(3H)-quinazolinone derivatives. BMC Res. Notes.

[10] Amir M., Ali I., Hassan M.Z. (2013). Design and synthesis of some new quinazolin-4-(3H)-ones as anticonvulsant and antidepressant agents. Arch. Pharm. Res..

[11] Frishman W.H., Kotob F. (1999). Alpha-adrenergic blocking drugs in clinical medicine. J. Clin. Pharmacol..

[12] Sica D.A. (2005). Alpha1-adrenergic blockers: Current usage considerations. J. Clin. Hypertens. (Greenwich Conn.).

[13] Gawad N.M.A., Georgey H.H., Youssef R.M., El-Sayed N.A. (2010). Synthesis and antitumor activity of some 2,3-disubstituted quinazolin-4(3H)-ones and 4,6-disubstituted-1,2,3,4-tetrahydroquinazolin-2H-ones. Eur. J. Med. Chem..

[14] Shagufta S., Ahmad I. (2017). An insight into the therapeutic potential of quinazoline derivatives as anticancer agents. Med. Chem. Commun..

[15] Asif M. (2014). Chemical characteristics, synthetic methods, and biological potential of quinazoline and quinazolinone derivatives. Int. J. Med. Chem..

[16] Khan I., Ibrar A., Ahmed W., Saeed A. (2015). Synthetic approaches, functionalization and therapeutic potential of quinazoline and quinazolinone skeletons: The advances continue. Eur. J. Med. Chem..

[17] Wang C., Sun Y., Zhu X., Wu B., Wang Q., Zhen Y., Shu X., Liu K., Zhou Y., Ma X. (2016). Novel quinazoline derivatives bearing various 4-aniline moieties as potent EGFR inhibitors with enhanced activity against NSCLC cell lines. Chem. Biol. Drug. Des..

[18] Faraj F.L., Zahedifard M., Paydar M., Looi C.Y., Abdul Majid N., Ali H.M., Ahmad N., Gwaram N.S., Abdulla M.A. (2014). Synthesis, characterization, and anticancer activity of new quinazoline derivatives against MCF-7 cells. Sci. World J..

[19] Cao S.L., Guo Y.W., Wang X.B., Zhang M., Feng Y.P., Jiang Y.Y., Wang X.B., Gao Q., Ren J. (2009). Synthesis and cytotoxicity screening of piperazine-1-carbodithioate derivatives of 2-substituted quinazolin-4(3H)-ones. Arch. Pharm..

[20] Marzaro G., Guiotto A., Chilin A. (2012). Quinazoline derivatives as potential anticancer agents: A patent review (2007–2010). Expert Opin. Ther. Pat..

[21] Tiwary K.B., Pradhan K., Nanda A.K., Chakraborty R. (2015). Implication of quinazoline-4(3H)-ones in medicinal chemistry: A Brief Review. J. Chem. Biol. Ther..

[22] Hameed A., Al-Rashida M., Uroos M., Ali S.A., Arshia I.M., Khan K.M. (2018). Quinazoline and quinazolinone as important medicinal scaffolds: A comparative patent review (2011–2016). Expert Opin. Ther. Pat..

[23] Cadenas E., Davies K.J.A. (2000). Mitochondrial free radical generation, oxidative stress, and aging. Free Radic. Biol. Med..

[24] Halliwell B., Gutteridge J.M.C. (2007). Free Radicals in Biology and Medicine.

[25] Sharma N. (2014). Free radicals, antioxidants and disease. Biol. Med..

[26] Thomas C., Mackey M.M., Diaz A.A., Cox D.P.C. (2009). Hydroxyl radical is produced via the Fenton reaction in submitochondrial particles under oxidative stress: Implications for diseases associated with iron accumulation. Redox Rep..

[27] Koppenol W.H. (2001). The Haber-Weiss cycle-70 years later. Redox Rep..

[28] Klaunig J., Kamendulis L. (2004). The role of oxidative stres in carcinogenesis. Ann. Rev. Pharmacol. Toxicol..

[29] Fang J., Seki T., Maeda H.J. (2009). Therapeutic strategies by modulating oxygen stress in cancer and inflammation. Adv. Drug Deliv. Rev..

[30] Ighodaro O.M., Akinloye O.A. (2018). First line defence antioxidants-superoxide dismutase (SOD), catalase (CAT) and glutathione peroxidase (GPX): Their fundamental role in the entire antioxidant defence grid. Alex. J. Med..

[31] Kurutas E.B. (2016). The importance of antioxidants which play the role in cellular response against oxidative/nitrosative stress: Current state. Nutr. J..

[32] Hricovíniová Z., Hricovíni M., Kozics K. (2018). New series of quinazolinone derived Schiff’s bases: Synthesis, spectroscopic properties and evaluation of their antioxidant and cytotoxic activity. Chem. Pap..

[33] Kuntikana S., Bhat C., Kongot M., Bhat I., Kumar A. (2016). An expeditious green cascade synthesis of 3-arylideneaminoquinazolin-4(1H)-one derivatives via ‘solvent drop grinding’ and their antioxidant and DNA protective studies. ChemistrySelect.

[34] Refaie F.M., Esmat A.Y., Gawad S.M.A., Ibrahim A.M., Mohamed M.A. (2005). The antihyperlipidemic activities of 4(3H) quinazolinone and two halogenated derivatives in rats. Lipids Health Dis..

[35] Zhang J., Liu J., Ma Y., Ren D., Cheng P., Zhao J., Zhang F., Yao Y. (2016). One-pot synthesis and antifungal activity against plant pathogens of quinazolinone derivatives containing an amide moiety. Bioorg. Med. Chem. Lett..

[36] He D., Wang M., Zhao S., Shu Y., Zeng H., Xiao C., Lu C., Liu Y. (2017). Pharmaceutical prospects of naturally occurring quinazolinone and its derivatives. Fitoterapia.

[37] Yang L., Gong J., Wang F., Zhang Y., Wang Y., Hao X., Wu X., Bai H., Stöckigt J., Zhao Y. (2006). Synthesis and antioxidant evaluation of novel silybin analogues. J. Enzym. Inhib. Med. Chem..

[38] Hricovíniová J., Ševčovičová A., Hricovíniová Z. (2020). Evaluation of the genotoxic, DNA-protective and antioxidant profile of synthetic alkyl gallates and gallotannins using in vitro assays. Toxicol. Vitr..

[39] Kozics K., Srančíková A., Sedláčková E., Horváthová E., Melušová M., Meluš V., Krajcovičová Z., Šramková M. (2017). Antioxidant potential of essential oil from Lavandula angustifolia in in vitro and ex vivo cultured liver cells. Neoplasma.

[40] Kumar A., Sharma P., Kumari P., Lal L., Kalal B. (2011). Exploration of antimicrobial and antioxidant potential of newly synthesized 2,3-disubstituted quinazoline-4(3H)-ones. Bioorg. Med. Chem. Lett..

[41] Glube N., Giessl A., Wolfrum U., Langguth P. (2007). Caki-1 cells represent an in vitro model system for studying the human proximal tubule epithelium. Nephron Exp. Nephrol..

[42] Zahedifard M., Faraj F.L., Paydar M., Looi C.Y., Hajrezaei M., Hasanpourghadi M., Kamalidehghan B., Majid N.A., Ali H.M., Abdulla M.A. (2015). Synthesis, characterization and apoptotic activity of quinazolinone Schiff base derivatives toward MCF-7 cells via intrinsic and extrinsic apoptosis pathways. Sci. Rep..

[43] Hassanzadeh F., Sadeghi-Aliabadi H., Jafari E., Sharifzadeh A., Dana N. (2019). Synthesis and cytotoxic evaluation of some quinazolinone- 5-(4-chlorophenyl) 1, 3, 4-oxadiazole conjugates. Pharm. Sci..

[44] Nallathamby N., Phan C.W., Sovan M., Saso L., Sabaratnam V. (2019). Synthesized 2-trifluoromethylquinazolines and quinazolinones protect BV2 and N2a cells against LPS- and H_2_O_2_-induced cytotoxicity. Med. Chem..

[45] Rakesh K.P., Kumara H.K., Manukumar H.M., Gowda D.C. (2019). Anticancer and DNA binding studies of potential amino acids based quinazolinone analogs: Synthesis, SAR and molecular docking. Bioorg. Chem..

[46] El-Sayed N.N.E., Alamneai N.M., Ben Bacha A., Al Obeed O., Ahmad R. (2019). Synthesis and evaluation of anticancer, antiphospholipases, antiproteases, and antimetabolic syndrome activities of some 3H-quinazolin-4-one derivatives. J. Enzym. Inhib. Med. Chem..

[47] Weidinger A., Kozlov A.V. (2015). Biological activities of reactive oxygen and nitrogen species: Oxidative stress versus signal transduction. Biomolecules.

[48] Suzuki K. (2009). Anti-oxidants for therapeutic use: Why are only a few drugs in clinical use?. Adv. Drug Deliv. Rev..

[49] Zhao F., Liu Z.Q. (2009). The protective effect of hydroxyl-Substituted Schiff bases on the radical-Induced oxidation of DNA. J. Phys. Org. Chem..

[50] Hossain S.U., Bhattacharya S. (2007). Synthesis of O-prenylated and O-geranylated derivatives of 5-benzylidene2,4-thiazolidinediones and evaluation of their free radical scavenging activity as well as effect on some phase II antioxidant/detoxifying enzymes. Bioorg. Med. Chem. Lett..

[51] Gudasi K.B., Patil S.A., Vadavi R.S., Shenoy R.V., Nethaji M. (2006). Crystal structure of 2-[2-hydroxy-3-methoxyphenyl]-3-[2-hydroxy-3-methoxybenzylamino]-1,2-dihydroquinazolin-4(3H)-one and the synthesis, spectral and thermal investigation of its transition metal complexes. Trans. Metal Chem..

[52] Locatelli M., Gindro R., Travaglia F., Coïsson J.D., Rinaldi M., Arlorio M. (2009). Study of the DPPH•-scavenging activity: Development of a free software for the correct interpretation of data. Food Chem..

[53] Gupta D. (2015). Methods for determination of antioxidant capacity: A review. Int. J. Pharm. Sci. Res..

[54] Čipák L., Miadoková E., Dingová H., Kogan G., Novotný L., Rauko P. (2001). Comparative DNA protectivity and antimutagenicity studies using DNA-topology and Ames assays. Toxicol. Vitro.

[55] Mosmann T. (1983). Rapid colorimetric assay for cellular growth and survival: Application to proliferation and cytotoxicity assays. J. Immunol. Methods.

[56] Singh N.P., McCoy M.T., Tice R.R., Schneider E.L. (1988). A simple technique for quantitation of low levels of DNA damage in individual cells. Exp. Cell Res..

[57] Gupta R., Sharma M., Lakshmy R., Prabhakaran D., Reddy K.S. (2009). Improved method of total antioxidant assay. Indian J. Biochem. Biophys..

[58] Paglia D.E., Valentine W.N. (1967). Studies on the quantitative and qualitative characterization of erythrocyte glutathione peroxidase. J. Lab. Clin. Med..

[59] Góth L. (1991). A simple method for determination of serum catalase activity and revision of reference range. Clin. Chim. Acta.

[60] Bradford M.M. (1976). A rapid and sensitive method for the quantitation of microgram quantities of protein utilizing the principle of protein-dye binding. Anal. Biochem..

